# Supplementary material for: Exploring the Tumor-Suppressing Potential of PSCA in Pancreatic Ductal Adenocarcinoma
Source: Cancers (Basel). 2023 Oct 10;15(20):4917. doi: 10.3390/cancers15204917 (PMC10605218; doi:10.3390/cancers15204917)
Supplement: Supplementary file 1 [file cancers-15-04917-s001.zip › cancers-2616817-supplementary.pdf]

Supplementary Information

Supplementary Table S1. Hydrogen-bonding interaction in MSLN-PSCA complex.

MSLN Receptor Residue	PSCA Ligand Residue	Interaction Constituents	Distance (Å)	Type
M:ASP464	B:LYS18	B:LYS18:NZ - M:ASP464:O	2.4	Conventional
M:ASP466	B:SER54	B:SER54:OG - M:ASP466:OD2	2.5	Conventional
M:ASP469	B:ASN60	B:ASN60:N - M:ASP469:OD2	2.9	Conventional
M:GLY439	B:SER16	M:GLY439:H - B:SER16:O	2.6	Conventional
M:GLY439	B:GLN28	M:GLY439:H - B:GLN28:OE1	2.7	Conventional
M:SER445	B:THR33	M:SER445:H - B:THR33:O	2.4	Conventional
M:THR467	B:CYS17	M:THR467:HG1 - B:CYS17:O	2.8	Conventional
M:THR467	B:LYS55	M:THR467:HG1 - B:LYS55:O	2.8	Conventional
M:GLN472	B:CYS57	M:GLN472:HE22 - B:CYS57:O	2.7	Conventional
M:ARG557	B:TYR68	M:ARG557:H - B:TYR68:OH	2.5	Conventional
M:ARG557	B:TYR68	M:ARG557:HE - B:TYR68:OH	1.8	Conventional
M:THR467	B:SER54	B:SER54:CB - M:THR467:OG1	2.9	Carbon
M:GLU406	B:LEU27	M:GLU406:HA - B:LEU27:O	2.0	Carbon
M:TYR437	B:GLN28	M:TYR437:HA - B:GLN28:OE1	1.9	Carbon
M:PRO438	B:GLN28	M:PRO438:HD2 - B:GLN28:OE1	1.7	Carbon
M:GLY439	B:SER16	M:GLY439:HA3 - B:SER16:OG	2.5	Carbon
M:SER443	B:SER16	M:SER443:HB3 - B:SER16:OG	2.1	Carbon
M:SER445	B:GLU37	M:SER445:HA - B:GLU37:OE2	2.1	Carbon
M:PRO446	B:GLU37	M:PRO446:HD3 - B:GLU37:OE2	2.5	Carbon
M:HIS556	B:GLN66	M:HIS556:HA - B:GLN66:OE1	2.4	Carbon
M:PRO558	B:TYR68	M:PRO558:HD3 - B:TYR68:OH	2.9	Carbon

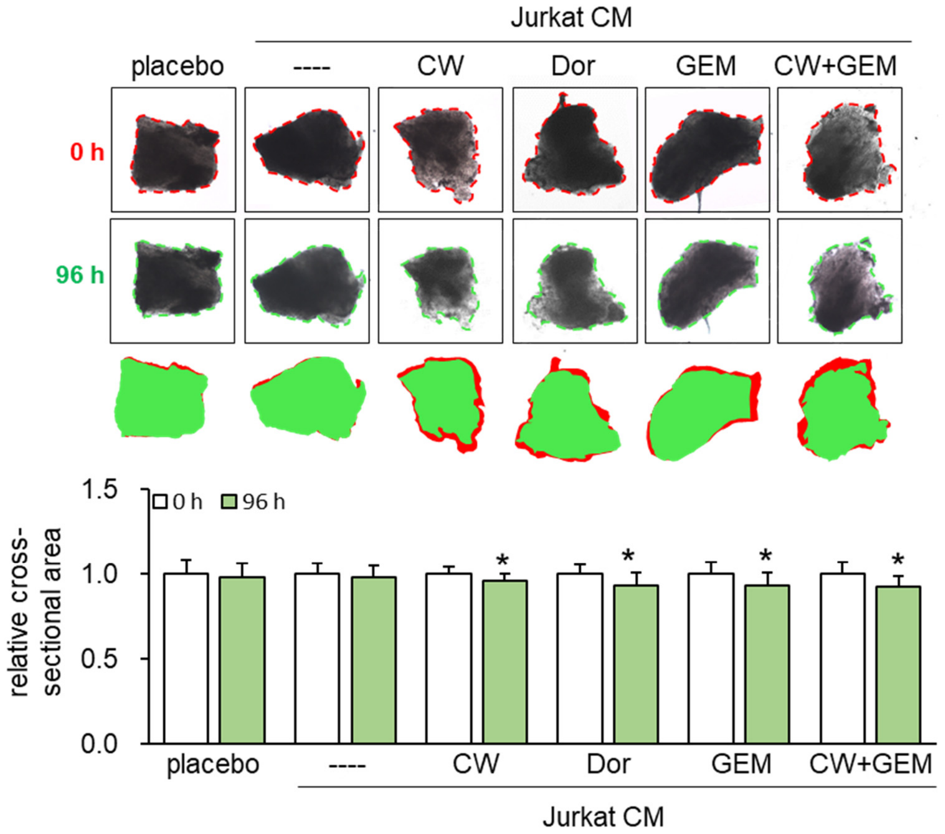

Supplementary Figure S1. Efficacy in the freshly isolated pancreatic cancer tissue fragments. The single asterisk indicates $p < 0.05$. CM = conditioned medium, CN = control, CW = CW008 as a PKA activator, Dor = Dorsomorphin as an AMPK inhibitor, and GEM = Gemcitabine. Diminished pancreatic cancer tissue fragments in 96 h by CW008 and Dorsomorphin-treated lymphocyte-derived CM with and without the co-application of Gemcitabine.
