# Supplementary material for: Exploring the Tumor-Suppressing Potential of PSCA in Pancreatic Ductal Adenocarcinoma
Source: Cancers (Basel). 2023 Oct 10;15(20):4917. doi: 10.3390/cancers15204917 (PMC10605218; doi:10.3390/cancers15204917)
Supplement: Supplementary file 1 [file cancers-15-04917-s001.zip › cancers-2616817-The original western blot figures.pdf]

Figure 2E

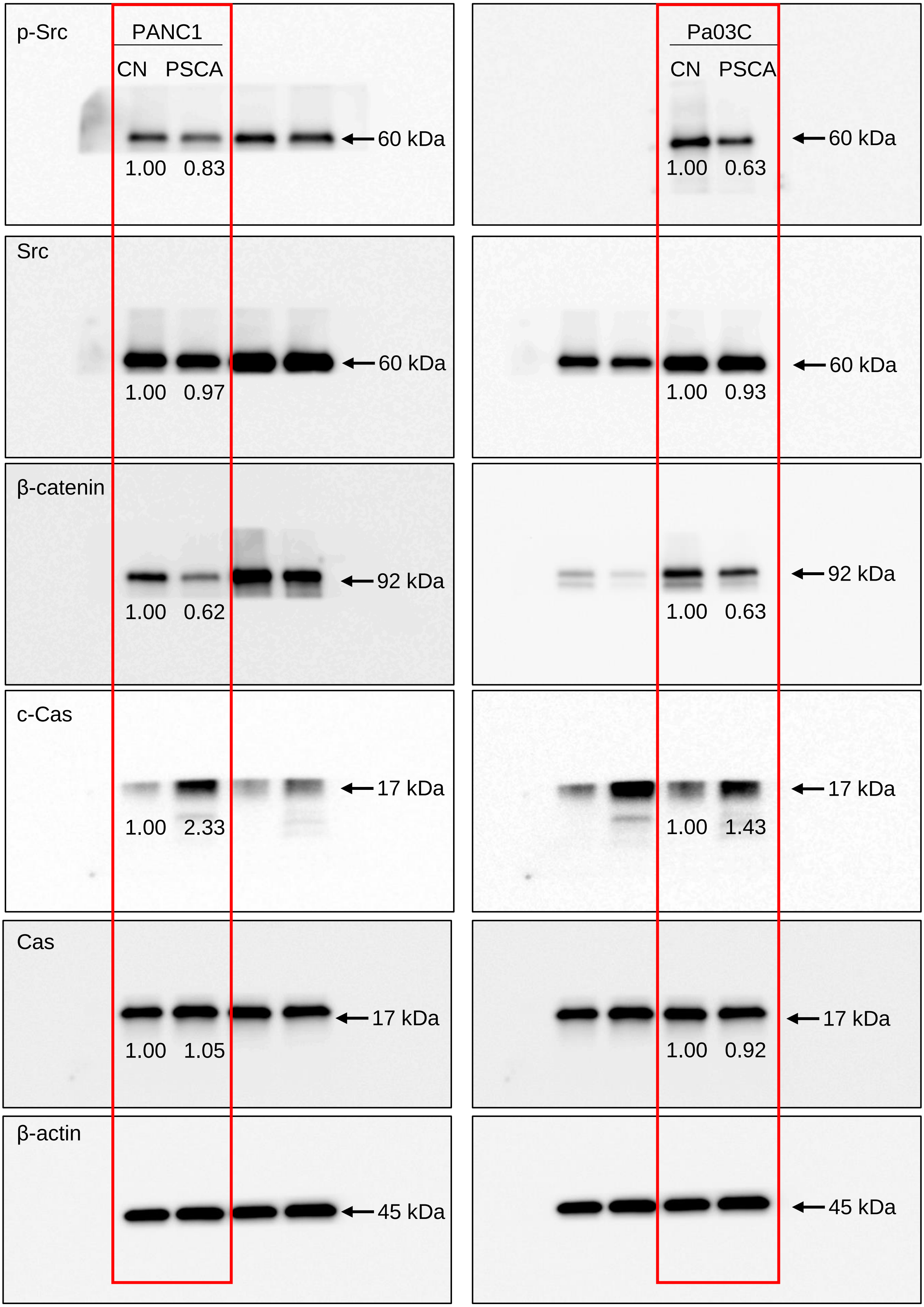

PANC1
CN PSCA
p-Src
60 kDa
1.00 0.83
Src
60 kDa
1.00 0.97
β-catenin
92 kDa
1.00 0.62
c-Cas
17 kDa
1.00 2.33
Cas
17 kDa
1.00 1.05
β-actin
45 kDa
Pa03C
CN PSCA
60 kDa
1.00 0.63
60 kDa
1.00 0.93
92 kDa
1.00 0.63
17 kDa
1.00 1.43
17 kDa
1.00 0.92
45 kDa

Figure 2F

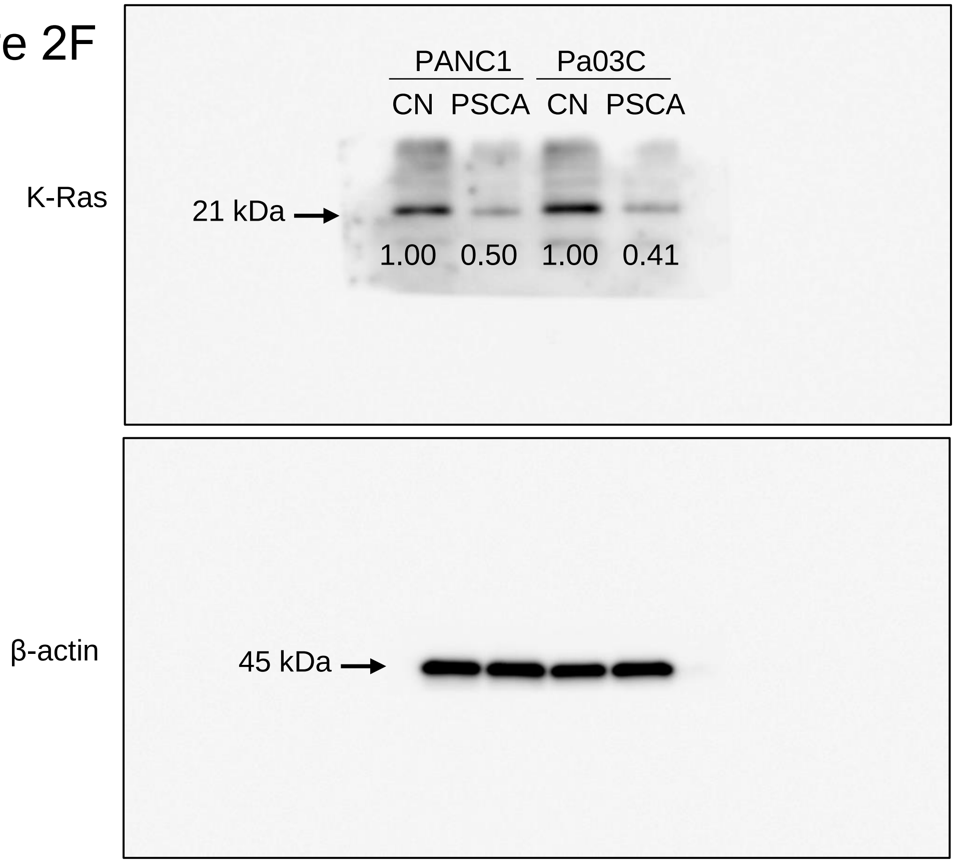

Figure 3D

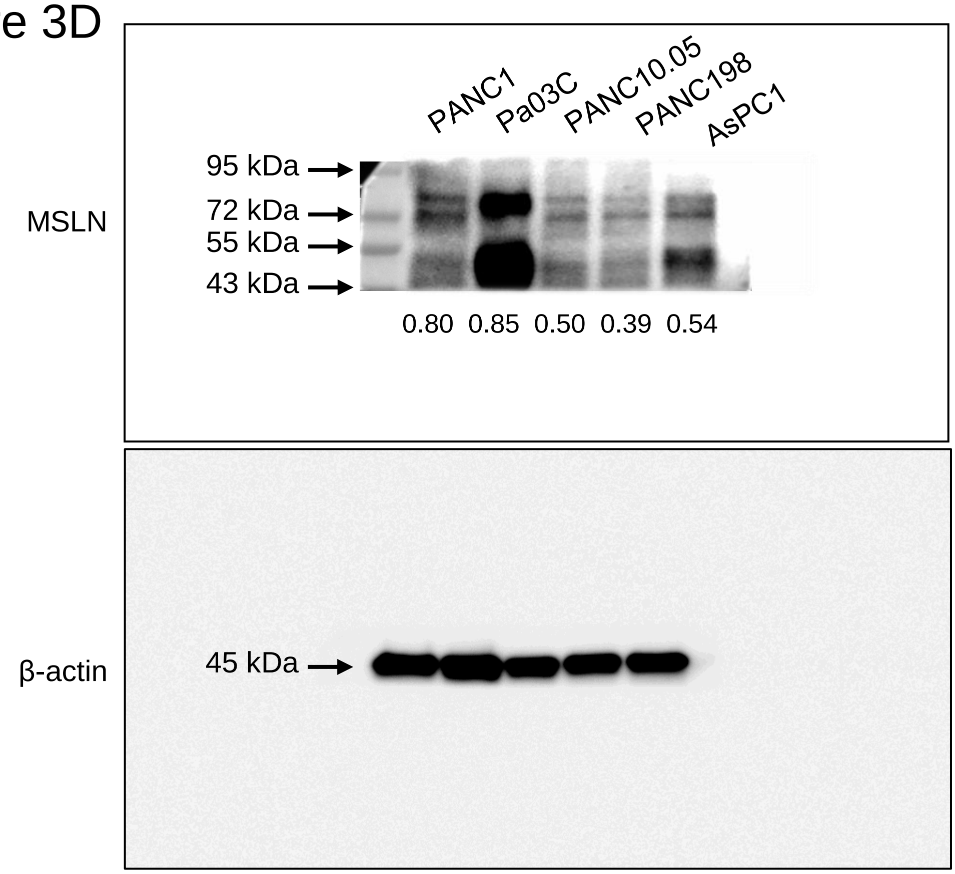

Figure 4B

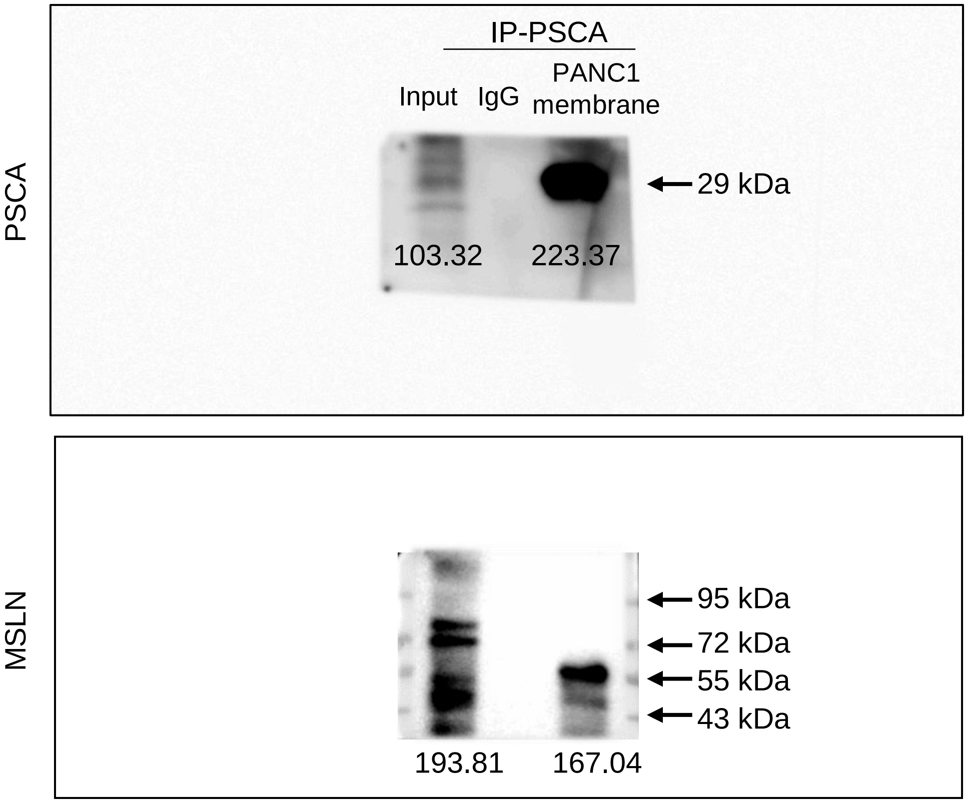

Figure 4C

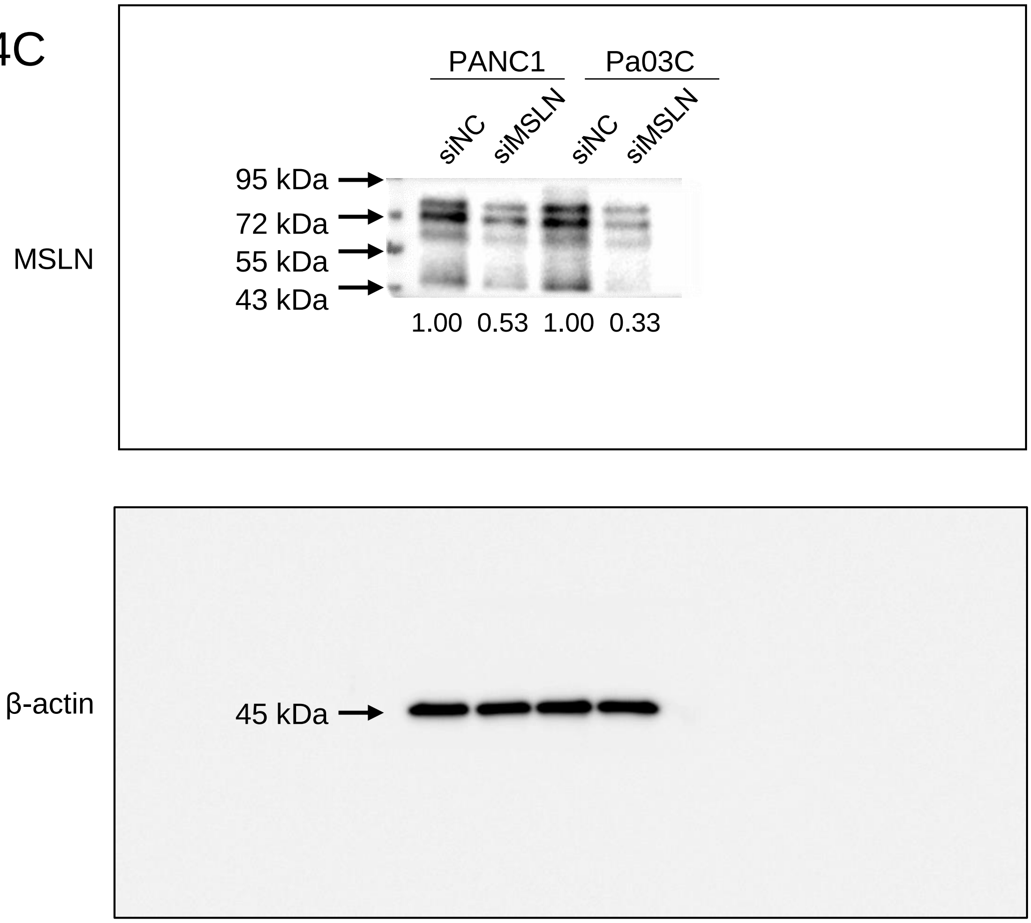

Figure 4E

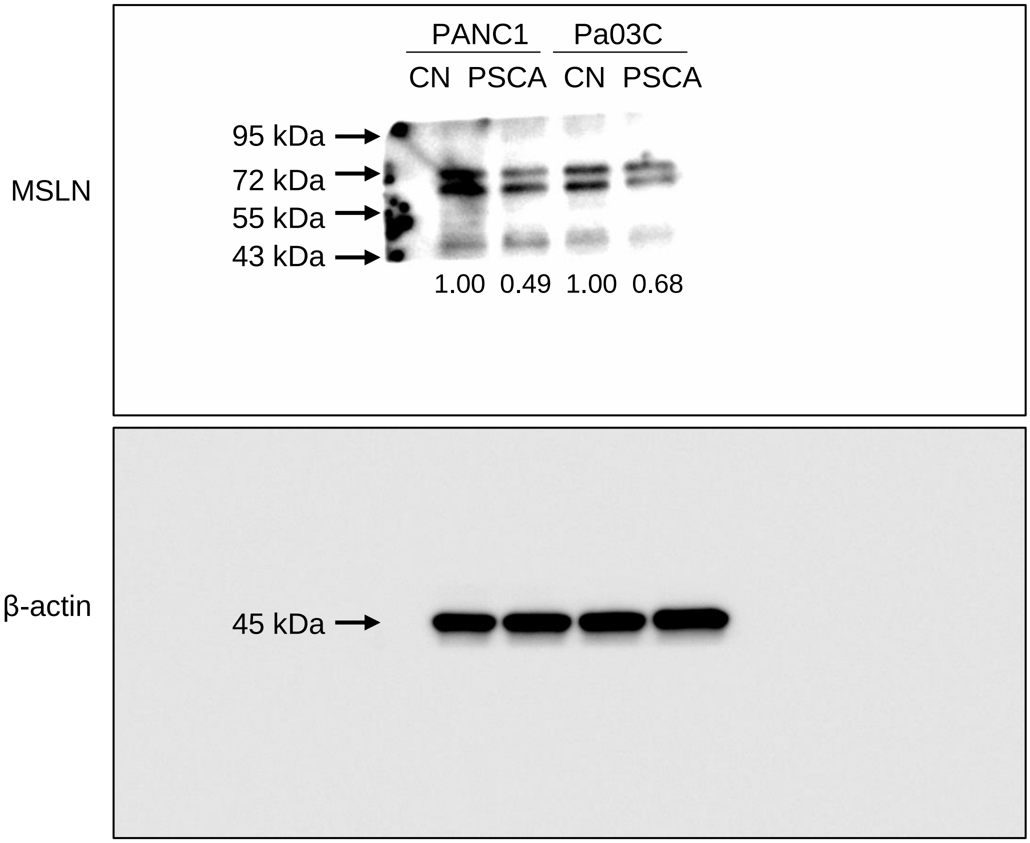
PANC1
Pa03C
CN
PSCA
CN
PSCA
MSLN
95 kDa
72 kDa
55 kDa
43 kDa
1.00
0.49
1.00
0.68
β-actin
45 kDa

Figure 6D

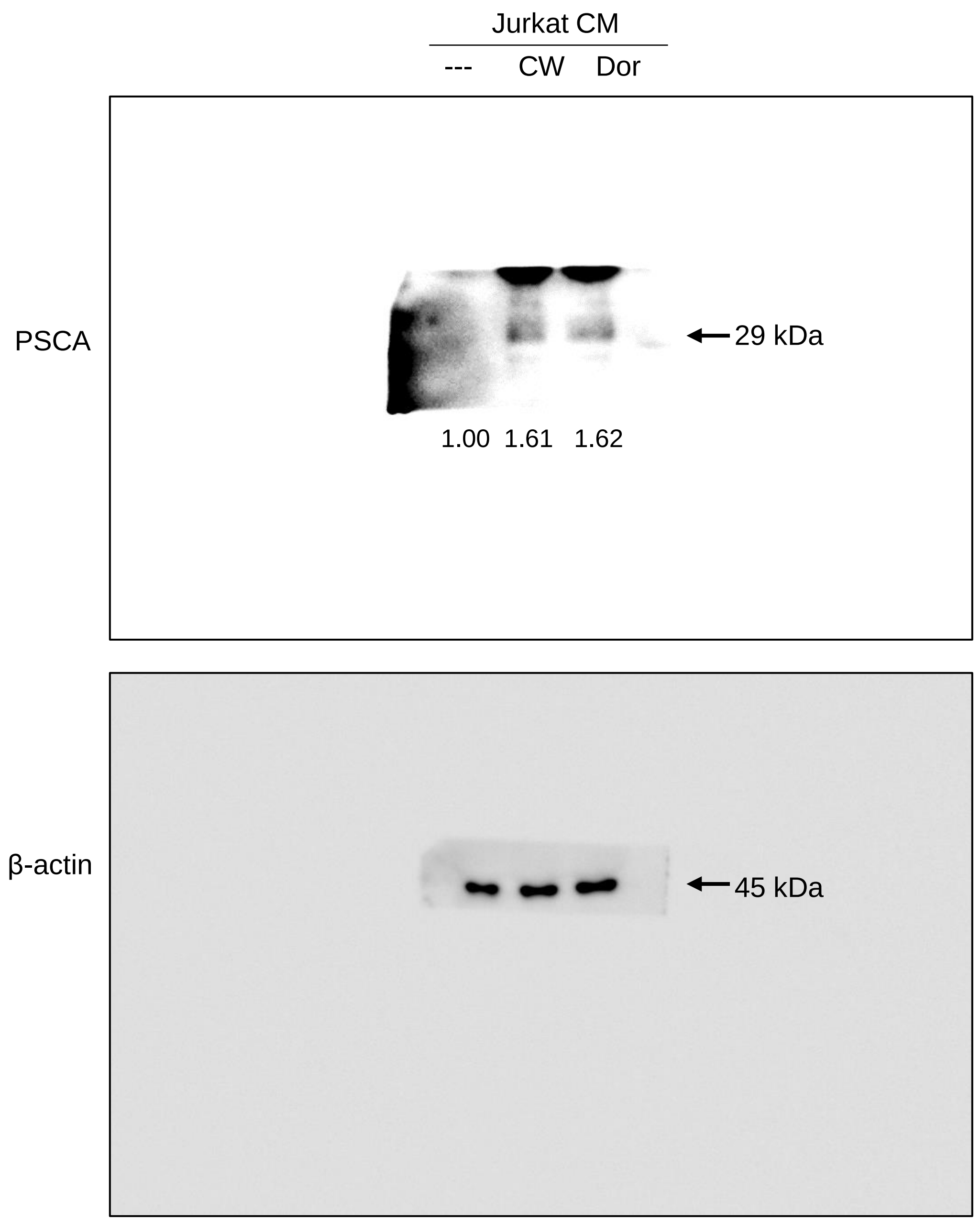

Figure 7C

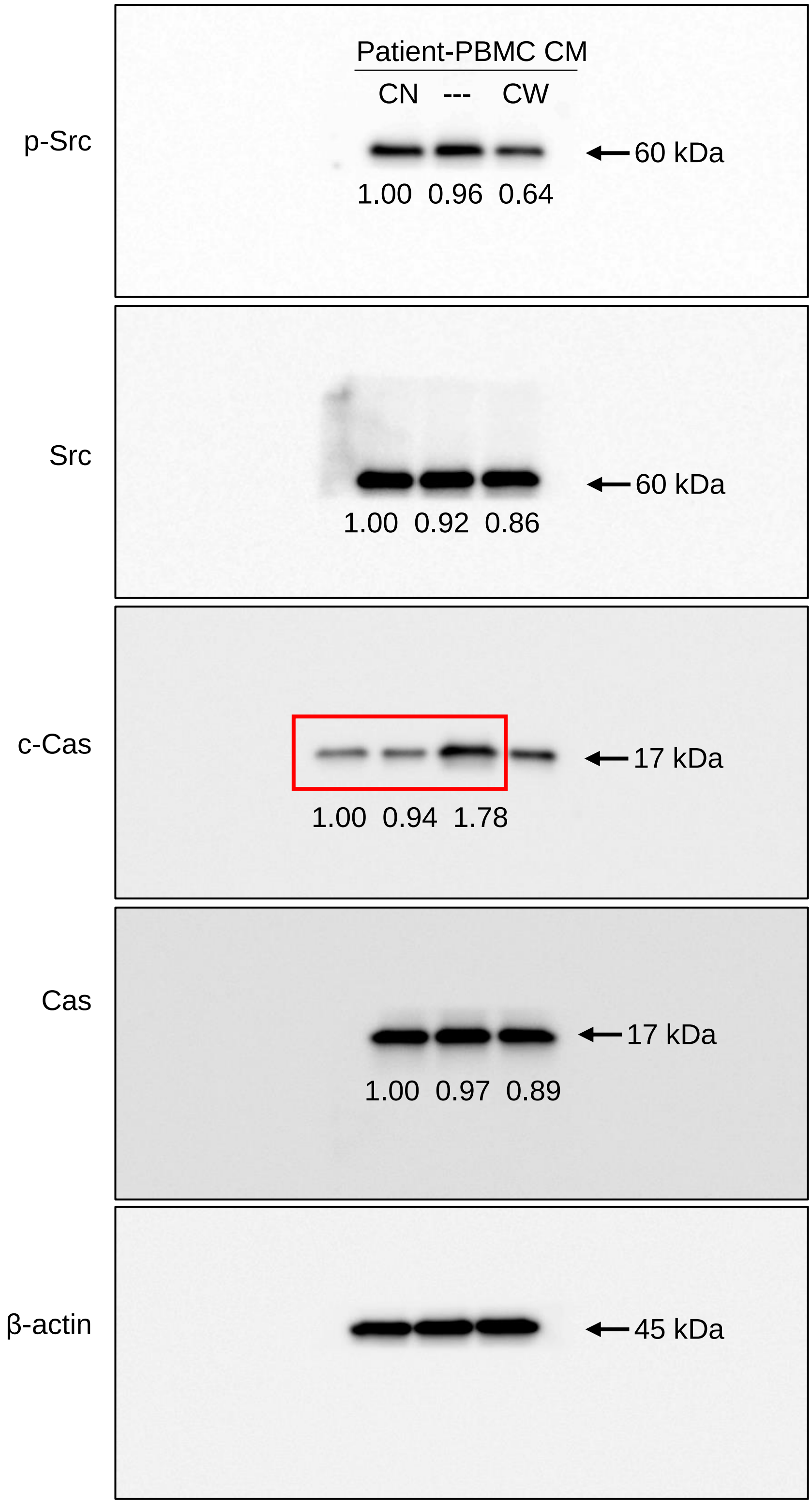

Figure S2. The original western blot figures.
